# Body Condition Indices Predict Reproductive Success but Not Survival in a Sedentary, Tropical Bird

**DOI:** 10.1371/journal.pone.0136582

**Published:** 2015-08-25

**Authors:** Olga Milenkaya, Daniel H. Catlin, Sarah Legge, Jeffrey R. Walters

**Affiliations:** 1 Department of Biological Sciences, Virginia Tech, Blacksburg, Virginia, United States of America; 2 Department of Fish and Wildlife Conservation, Virginia Tech, Blacksburg, Virginia, United States of America; 3 Australian Wildlife Conservancy, Mornington Wildlife Sanctuary, Derby, Western Australia, Australia; 4 Research Institute for Environment and Livelihoods, Charles Darwin University, Casuarina, Northern Territory, Australia; Phillip Island Nature Parks, AUSTRALIA

## Abstract

Body condition may predict individual fitness because those in better condition have more resources to allocate towards improving their fitness. However, the hypothesis that condition indices are meaningful proxies for fitness has been questioned. Here, we ask if intraspecific variation in condition indices predicts annual reproductive success and survival. We monitored a population of *Neochmia phaeton* (crimson finch), a sedentary, tropical passerine, for reproductive success and survival over four breeding seasons, and sampled them for commonly used condition indices: mass adjusted for body size, muscle and fat scores, packed cell volume, hemoglobin concentration, total plasma protein, and heterophil to lymphocyte ratio. Our study population is well suited for this research because individuals forage in common areas and do not hold territories such that variation in condition between individuals is not confounded by differences in habitat quality. Furthermore, we controlled for factors that are known to impact condition indices in our study population (e.g., breeding stage) such that we assessed individual condition relative to others in the same context. Condition indices that reflect energy reserves predicted both the probability of an individual fledging young and the number of young produced that survived to independence, but only during some years. Those that were relatively heavy for their body size produced about three times more independent young compared to light individuals. That energy reserves are a meaningful predictor of reproductive success in a sedentary passerine supports the idea that energy reserves are at least sometimes predictors of fitness. However, hematological indices failed to predict reproductive success and none of the indices predicted survival. Therefore, some but not all condition indices may be informative, but because we found that most indices did not predict any component of fitness, we question the ubiquitous interpretation of condition indices as surrogates for individual quality and fitness.

## Introduction

Predicting individual fitness of wild organisms is important because intraspecific variation in fitness has broad implications for evolution and higher ecological processes within populations and communities. Researchers are therefore interested in measures that may be meaningful proxies for fitness and predict individual differences in reproductive success and survival. One such proxy is body condition (hereafter “condition”). Condition is most often defined as the pool of resources that an individual has acquired (and presumably assimilated) that can be allocated toward improving fitness [1,2,3], but see [4] for a different definition. Condition is most informative when it is employed in a relative sense to compare individuals within populations or between places and times. In such contexts the pool of available resources (condition) traditionally has been expected to predict fitness since those with more resources can allocate more toward improving their realized fitness. This condition-fitness relationship may be mediated by individual quality because those of superior quality are expected to be better at acquiring and using resources, thereby improving their fitness. Whether related to individual quality or not, those in better condition are expected to have higher fitness.

There is no direct way to test whether condition predicts fitness because condition is not directly quantifiable. Researchers instead use condition indices as its surrogate. These indices reflect different aspects of an animal’s physiology including resource acquisition and allocation, nutritional status, immunocompetence and stress. We emphasize that condition indices are not the same as condition [5], but are instead tools for understanding aspects of condition.

Because condition indices measure condition and condition is expected to predict fitness, condition indices are often interpreted as indicators of individual quality and fitness. Indeed, some condition indices have been demonstrated to predict reproductive traits [6,7] and survival [8,9,10], but the common interpretation of condition indices as proxies for fitness has been questioned [11,12] and condition indices remain poorly validated. Here, we test whether condition indices are meaningful proxies for fitness by asking if seven commonly-used condition indices predict survival and annual reproductive success.

There are two main challenges to testing whether condition indices are meaningful proxies for fitness: first, condition indices vary by context (e.g. breeding stage) such that differences in condition indices may reflect the context rather than individual condition *per se*. Because we have previously determined how condition indices in our study population vary by sex, breeding stage, age, year, and time of day [13], we can control for this variation and assess individual condition relative to others in the same context. Second, while condition is expected to be related to fitness, it is also subject to extrinsic factors such as the quality of the environment [14,15,16]. This makes it difficult to infer fitness from condition where animals have access to habitats of varying quality such as in species that hold territories. However, in our study system we evaluate variation in condition largely independent of habitat quality because individuals are not territorial [17] and forage in common areas within a small study area (O. Milenkaya, *personal observation*). We acknowledge that other confounding factors such as social structure may influence condition in our study population but we expect these effects to be weaker than those caused by differences in habitat quality in territorial species. By assessing condition indices relative to others in the same context and environment, we are able to avoid confounding factors found in previous field studies. Our aim is to use this unique opportunity to comprehensively answer the question: are condition indices meaningful predictors of fitness?

## Materials and Methods

### Ethics statement

We made all efforts to minimize animal suffering and our methods were approved by the Animal Ethics Committee of the Department of Conservation and Land Management, Western Australia (CAEC/6/2005 and DEC AEC 43/2007).

### Study species and study area

*Neochmia phaeton* (Family Estrildidae) are small (~10 g) passerines occupying riparian areas in northern Australia and New Guinea [18]. They breed as socially monogamous pairs in which males build the nests, females lay clutches of one to seven eggs (average = five), and both sexes incubate the eggs, and provision and defend the young [17]. We studied a population of wild *Neochmia phaeton* at Mornington Wildlife Sanctuary in northwest Australia (17°30&rsquo;49ʺ S, 126°06&rsquo;39ʺ E, 200 masl), a private land-holding of the Australian Wildlife Conservancy. Our study area consisted of riparian habitat along a two kilometer stretch of creek. Individual birds were monitored for reproductive success and survival, and sampled for condition indices, over four consecutive breeding seasons (15 November 2006–11 April 2007, 5 December 2007–31 May 2008, 9 December 2008–29 May 2009, and 10 December 2009–10 May 2010).

Our study population is well suited for this research because individuals vary in both reproductive success [17] and condition indices [13]. Furthermore, nests and fledglings are readily detected, which allowed us to document reproductive success with relative accuracy. Because the birds are sedentary, conspicuous and restricted to a narrow habitat zone, recapture probabilities are high (89−93%; [17]) and therefore estimates of survival are relatively precise.

### General field methods

We captured adult *Neochmia phaeton* in mist-nets and banded them with a metal band (Australian Bird and Bat Banding Scheme) and up to three plastic color bands for individual recognition. We determined the sex of each bird based on their sexually dimorphic plumage [18].

We monitored the breeding attempts of individual birds and identified birds as being in one of the following stages of the breeding cycle at the time of capture: pre-breeding, nest building, egg laying, incubating, nestling, or post-breeding. *Neochmia phaeton* in our study area are multi-brooded [17], so the nest building, egg laying, incubating, and nestling stages occur throughout the breeding season and do not necessarily represent the first breeding attempt of the season. The pre and post-breeding stages, however, correspond to the beginning and end of the breeding seasons, respectively.

### Condition indices

We sampled birds for both traditional condition indices that reflect energy reserves (mass adjusted for body size, and muscle and fat scores) as well as hematological parameters that reflect other aspects of condition. Although hematological indices do not measure energy reserves they measure aspects of physiology that may be related to fitness and are often considered condition indices. The measured indices are described in detail in Milenkaya et al. [13], but we briefly summarize them here. We weighed birds, measured the distance from the tip of the beak to the back of the head as a measure of structural size, and from these measures calculated a scaled mass index (scaled mass (g)) as a measure of mass corrected for size following Peig and Green [19]. We scored the amount of pectoral muscle around the keel bone (muscle score) and the amount of fat in the furcular (fat score) as described in Milenkaya et al. [13].

To sample for hematological condition indices, we punctured the birds’ brachial vein with a needle, collected ~5 μL of blood in a cuvette and used the portable HemoCue Hb 201+ Analyzer (HemoCue, Inc., Cypress, CA, USA) in the field to estimate hemoglobin concentration from whole blood (hemoglobin (g/L)). We collected additional whole blood (up to 40 μL) into plastic, sodium-heparinized micro-hematocrit capillary tubes, plugged the tubes and stored them on ice. Within a few hours, we centrifuged (Hettich Haematokrit 210) the capillary tubes for fifteen minutes at 1433.6 × g and read the proportion of red blood cells to the total volume of blood (packed cell volume (%)). During 2008−2009 and 2009−2010, we also extracted plasma from capillary tubes after centrifuging, and read the total plasma protein value (g/dL) with a hand-held refractometer (HR-200 ATC refractometer, AFAB Enterprises, Eustis, Florida, USA).

We sampled for H/L ratio during 2008−2009 and 2009−2010 by preparing blood smears in the field and fixing the slides in 100% methanol on the same day. Smears were later stained with Dip Quick Stain Set (Jorgensen Laboratories, Inc., Loveland, Colorado, USA) and one of us (OM) performed a leukocyte differential under 1000× magnification by identifying 100 leukocytes as a heterophil, lymphocyte, eosinophil, basophil, or monocyte. The H/L ratio was calculated as the relative proportion of heterophils to lymphocytes counted in the blood smear.

### Statistical analyses

We conducted the following analyses to determine if condition indices predict reproductive success and survival using (a) data from all four years of the study (4-year dataset), and (b) data from the latter two years of the study (2008−2009 and 2009−2010 breeding seasons; 2-year dataset). We conducted the 2-year analyses as well as the 4-year analyses because some condition indices (H/L ratio and total plasma protein) were only sampled in the latter two years of our study. Within each dataset the individual birds used in analyses of survival and reproductive success are not identical because we did not have reproductive success data on all individuals that were monitored for survival.

Our analyses of whether condition indices predict survival and reproductive success include several covariates that affect either the dependent variable or the condition indices of *Neochmia phaeton* in our study area [13]. These covariates include the birds’ sex (Sex), breeding stage at the time of condition sampling (Stage), age (Age), and the year (Year). All condition indices were approximately normally distributed except for H/L ratio, on which we performed a Log base-10 transformation. The condition indices were centered with a mean of zero (i.e., the intercept is now the mean of the values) before being analyzed.

### Principal components analysis

Indicators of individual quality may be less informative singularly than within a multivariate approach [**20**]. We therefore performed a principal components analysis using the correlation matrix for all condition indices within the 4-year and 2-year datasets for both the survival and reproductive success analyses. The 4-year datasets include packed cell volume, hemoglobin, scaled mass, muscle score and fat score, and the 2-year datasets additionally include H/L ratio and total plasma protein. We extracted the principal components (PCs) with an eigenvalue > 1 to use as additional condition indices and included them as explanatory variables in our models of survival and reproductive success. We conducted the PCAs in R version 3.0.0 [**21**].

### Survival and reproductive success analyses

We broadly approached our analysis of how condition indices may influence survival or reproductive success in a similar way. To reduce the number of models under consideration, we first identified the most relevant baseline model with a multiple step procedure (see below) [**22**], then built our models of condition indices upon the structure of these baseline models. To limit the number of models under consideration, we did not include combinations of condition indices. Instead, we included separate models for the principal components (see above) which incorporated information from all indices. We created a model for the additive effect of each condition index (including the PCs), and because condition indices may have non-linear effects on survival and reproductive success, we also considered additional models in which quadratic terms of the condition indices were included. We controlled for covariates known to affect condition indices in our study population [**13**] by including additional models where these individual covariates were included as additive effects with the condition indices that they impact. In summary, the candidate model sets included six types of models: (1) baseline, (2) baseline + condition, (3) baseline + condition + condition^2^, (4) baseline + condition + covariate(s), (5) baseline + condition + condition^2^ + covariate(s), and (6) the set of models that were included in the baseline model selection process (see below; S1–S3 Tables).

We used Akaike’s information criterion corrected for small sample sizes (AICc) and overdispersion (QAICc), model weights and evidence ratios (i.e. the ratio of model weights comparing two models) to evaluate the evidence for relationships of condition indices to reproductive success and survival. Where we found such evidence, we further assessed the impact of that condition index by calculating model-averaged predictions and presenting them with unconditional standard error. We used model-averaging because we had high model uncertainty, and we did so across the entire candidate model set. Where individual covariates were included in the baseline models, we evaluated the evidence for a relationship between the covariate(s) and reproductive success or survival. We elaborate on this elsewhere [23] and in the interest of brevity do not include these methods or results here but instead focus on the relationship between condition indices and reproductive success and survival.

### Reproductive success analyses

Approximately half of breeding pairs in our study area fail to fledge young during a given breeding season with predation being the primary cause of nest failure [**17**]. Because so many individuals have zero annual reproductive success, we asked first (1) do condition indices predict whether or not an individual will fledge young, and then (2) for those that do fledge at least one young, do their condition indices predict the number of young that they fledge? As *Neochmia phaeton* fledglings have only a 55% survival rate from fledging to independence [**17**], we also asked (3) whether condition indices of adults that successfully fledge at least one young predict the number of those young produced that survive to independence.

To address the first question, we built generalized linear mixed models with a binomial distribution and the logit link function where the response variable was whether (1) or not (0) the individual had fledged young in a given breeding season. To address the second and third questions, we ran the models with a poisson distribution and the log link function where the response variable was the number of young fledged and the number of young produced that survived to independence, respectively. In all of these analyses, the individual bird’s identity was used as a random factor because some individuals were sampled across breeding seasons. If an individual was sampled more than once for condition indices within a breeding season, then we randomly selected which sampling occasion to include for that year. The explanatory variables of interest in the models were the condition indices and principal components.

We determined the best baseline models by evaluating how Stage, Age, Year and, where feasible, their pairwise interactions affected reproductive success within each dataset. We did not include Sex as a covariate because we did not expect reproductive success to vary by sex.

We built our models of condition indices upon the structure of the baseline model and evaluated the evidence for them as previously described. We used R packages lme4 [24] and AICcmodavg [25].

**Fig 1:**
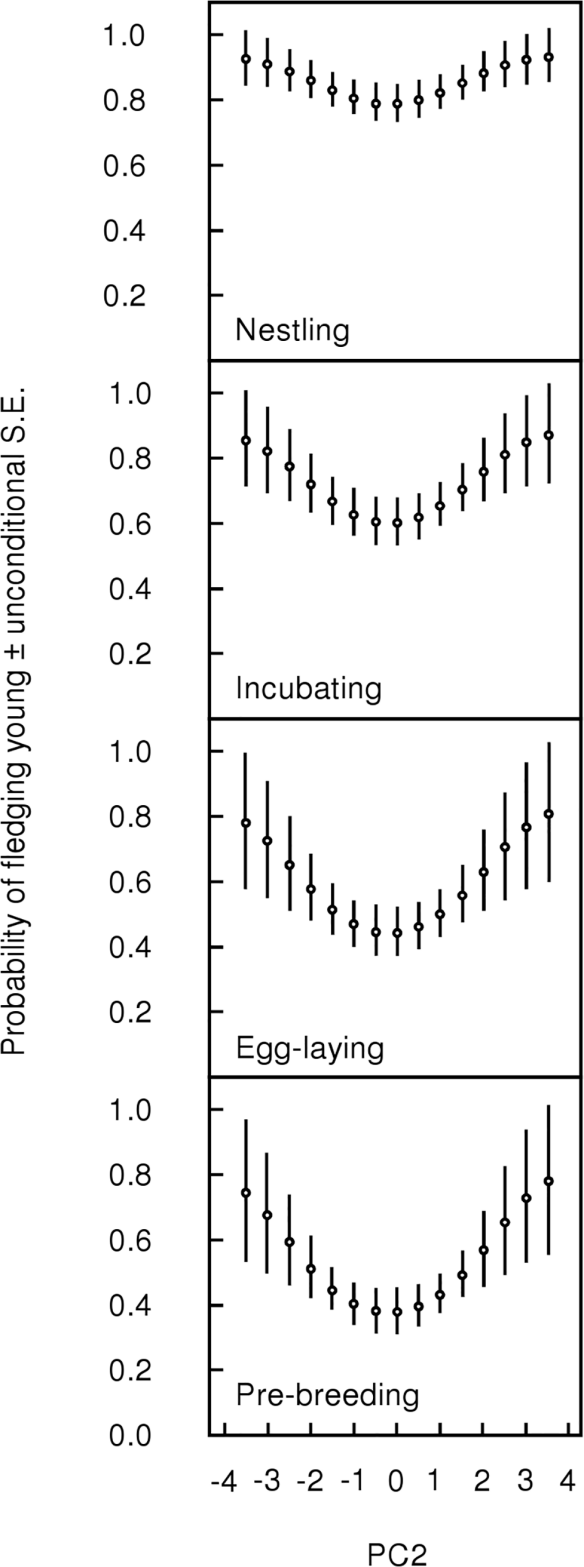
Relationship between PC2 and the probability of an adult fledging at least one young. PC2 is an axis of variation in individual condition indices (packed cell volume, hemoglobin, scaled mass, muscle score, fat score) with those having high energy reserves and high oxygen carrying capacity on the positive end of the axis, and those having low energy reserves and low oxygen carrying capacity on the negative end of the axis. Breeding stages refer to the stage of the adult when he/she was sampled for condition indices (pre-breeding, egg-laying, incubating, and nestling stages).

We performed post-hoc tests to explore possible explanations for an unexpected result in the relationship between PC2 and the probability of fledging young (see Results, Fig 1). We asked if individuals in the upper and lower quartiles of PC2 scores differ in age (first year versus after-first year); reproductive effort during the current breeding season including the number of nesting attempts, clutches laid, and broods hatched; reproductive effort and success in the previous breeding season including the number of nesting attempts, clutches laid, broods hatched, young fledged, and young produced that survive to independence; and survival of the bird to the following breeding season through band re-sighting. We used R version 3.0.0 [21] to conduct chi-square tests without continuity correction for age and survival data, and the Mann-Whitney-Wilcoxon test for the other variables. We set statistical significance at α = 0.05.

### Survival analyses

To test the effects of condition indices on apparent survival (φ) of adult *Neochmia phaeton*, we fit Cormack-Jolly-Seber models in Program MARK [**26**]. We used both recapture and resighting events (21 and 8 monthly occasions for the 4 and 2-year analyses, respectively) within our study area to build the encounter histories. Birds resighted opportunistically outside of the study site were excluded because they were known to have dispersed and were not systematically monitored for survival after dispersal (*n* = 10 individuals). We only included individuals for whom we had data on condition indices and their breeding stage at the time of sampling (*n* = 232 and 109 for the 4 and 2-year analyses, respectively), and we tested for apparent monthly survival following the occasion in which the individual was sampled for condition (rather than the occasion during which the individual was first banded). The datasets and analyses are summarized in Table **1**.

**Table 1:** Summary of survival analyses of the 4 and 2-year datasets.

	4-year analysis	2-year analysis
ĉ ± SE	1.21 ± 0.004	1.1 ± 0.006
Baseline model	ϕ(Sex+Age) p(*t*+Year+Sex+Stage)	ϕ(Year+Sex)p(*t*+Sex)
Packed cell volume	●	●
Hemoglobin	●	●
Scaled mass index	●	●
Muscle score	●	●
Fat score	●	●
Total plasma protein	NA	●
H/L ratio^a^	NA	●
PC1 ^b^	●	●
PC2 ^b^	●	●
PC3^b^	NA	●

Included are the estimated variance-inflation factor (ĉ ± SE), baseline model, and the condition indices included in the analysis (● = included, NA = not applicable).

^a^H/L ratio = heterophil to lymphocyte ratio.

^b^PCs = principal components.

We tested for goodness of fit by using the median ĉ test to estimate the variance-inflation factor (ĉ) for the fully time dependent model where both the apparent survival rate (ϕ) and the recapture probability (p) varied with time (ϕ(*t*) p(*t*)). Where appropriate, we adjusted for the median ĉ value and used QAICc thereafter in evaluating the evidence for our models.

In the multiple step procedure to determine the most relevant baseline model, we first evaluated the evidence for structural parameters (*t* and Year, where applicable) in both ϕ and p [22]. In the first step, we structured ϕ to be saturated with the structural parameters (ϕ(*t* +Year+(*t* × Year)) and compared alternate versions of p, testing all combinations of *t* and Year as well as a constant (‘.’) model. Having selected the best structure for p, we then compared alternate versions of ϕ in the same manner as for p, and selected the best structure for ϕ.

After developing a baseline model with structural components, we added the following nuisance covariates to control for their potential effects on both ϕ and p: Sex, Age, Sex × Age, and additionally for p also Stage, Sex × Stage, and Age × Stage. We did not consider Stage as a covariate for ϕ because we do not expect breeding stage to impact apparent survival. We compared models where combinations of these nuisance variables were added to the best structural model for p (with ϕ held constant at the best structural model), and, after selecting the best model for p, we repeated the process for ϕ (with p held constant at the best model including nuisance covariates). We excluded some covariates from our 4-year baseline model selection process because we lacked sufficient data to model them: Sex × Age for ϕ and Sex × Stage for p. At each step, if more than one model was competitive (within two delta AICc units of the top model) we selected the most parameterized model to proceed to the next step in an effort to explain the maximum amount of underlying variation. This process allowed us to narrow our candidate model set and to select the best baseline model (Table 1) for use as the foundation upon which our hypotheses of interest were tested.

We evaluated the evidence for our models using an information theoretic approach as previously described and we model-averaged to estimate ϕ and p (95% CI).

All data files are available from the Dryad database (DOI: doi:10.5061/dryad.3n2j5).

## Results

### Principal components analysis

We extracted two and three PCs from the 4 and 2-year datasets, respectively. In all cases, the first PC was primarily explained by a positive correlation between packed cell volume and hemoglobin (Table 2). The highest loading for the second PC in the 4-year dataset for both survival and reproductive success was muscle score. The second and third PCs varied between analyses of the 2-year dataset, but were consistent in involving those indices that most closely reflect energy reserves, namely fat, muscle and scaled mass (Table 2). Together, the two PCs of the 4-year datasets explained >63% of the total variance, and the three PCs of the 2-year datasets explained >65% of the total variance.

**Table 2:** PCA results for the reproductive success and survival analyses of both the 4 and 2-year datasets.

	4-year				2-year					
	survival		reproductive success		survival			reproductive success		
	PC1	PC2	PC1	PC2	PC1	PC2	PC3	PC1	PC2	PC3
Eigenvalue	1.85	1.3	1.91	1.28	2.14	1.4	1.09	2.26	1.21	1.12
Percent / cumulative	37/37	26/63	38/38	26/64	31/31	20/51	16/66	32/32	17/49	16/66
Packed cell volume	**-0.6**	0.37	**-0.6**	0.38	**0.57**	-0.13	0.3	**0.55**	-0.16	0.35
Hemoglobin	**-0.61**	0.31	**-0.6**	0.32	**0.55**	-0.21	0.36	**0.53**	-0.21	0.41
Scaled mass	0.37	0.46	0.36	0.39	-0.36	-0.5	0.06	-0.35	**-0.53**	0.08
Muscle score	0.16	**0.6**	0.19	**0.61**	0.09	**-0.63**	-0.1	0.19	-0.47	-0.25
Fat score	0.33	0.44	0.34	0.48	-0.26	-0.16	**0.66**	-0.28	-0.05	**0.67**
H/L ratio^a^	NA	NA	NA	NA	-0.08	0.49	0.49	0.01	**0.62**	0.28
Total plasma protein	NA	NA	NA	NA	-0.4	-0.15	0.31	-0.42	-0.2	0.34

Included are PCs with eigenvalues > 1, their eigenvalue, percent of variation in the data explained by the PC (percent), cumulative percent of variation explained by the PCs (cumulative), and loadings on each PC by the condition indices with the highest loadings in bold (NA = not applicable).

^a^H/L ratio = heterophil to lymphocyte ratio.

### Reproductive success

Complete AICc results are provided in S2 and S3 Tables. None of the condition indices predict whether an individual will fledge young in the 2-year dataset as evidenced by substantial model-uncertainty with the top model having only 11% of the weight (S3 Table), and the baseline model being within two delta AICc units of the top model. However, there is less model uncertainty in the larger 4-year dataset with the top model having 66% of the weight. This model includes a quadratic effect of PC2 and its evidence ratio is 13.2 against the baseline model, and 33.0 against the linear model of PC2. The effect of PC2 on the probability of fledging young is approximately parabolic around the mean, such that having an average PC2 score minimizes the probability of fledging young compared to above or below average PC2 scores that maximize the probability of successfully fledging at least one young (Fig 1).

Post-hoc tests comparing individuals in the lower and upper quartiles of PC2 scores indicate that the two groups did not differ in any variables available to us including age (X^2^(1, *n* = 146) = 1.71, *P* = 0.19); reproductive effort during the current breeding season such as number of nesting attempts (medians for both low and high PC2 groups was 3 nests, W = 3487, *n*_lower_ = 83, *n*_upper_ = 82, *P* = 0.78), clutches laid (medians for both low and high PC2 groups was 2 clutches, W = 2512, *n*_lower_ = 75, *n*_upper_ = 71, *P* = 0.54), and broods hatched (medians for both low and high PC2 groups was 1 brood, W = 2941.5, *n*_lower_ = 78, *n*_upper_ = 76, *P* = 0.93); reproductive effort and success in the previous breeding season such as number of nesting attempts (median for low and high PC2 groups were 2 and 3 nests, respectively, W = 367, *n*_lower_ = 43, *n*_upper_ = 23, *P* = 0.08), clutches laid (medians for low and high PC2 groups were 2 and 1 clutches, respectively, W = 293, *n*_lower_ = 33, *n*_upper_ = 18, *P* = 0.94), broods hatched (medians for both low and high PC2 groups was 1 brood, W = 266, *n*_lower_ = 39, *n*_upper_ = 16, *P* = 0.33), young fledged (medians for low and high PC2 groups were 0 and 1 young, respectively, W = 318, *n*_lower_ = 36, *n*_upper_ = 18, *P* = 0.91), and young produced that survive to independence (medians for both low and high PC2 groups was 0 young, W = 317.5, *n*_lower_ = 38, *n*_upper_ = 16, *P* = 0.76); or survival (X^2^(1, *n* = 130) = 0.01, *P* = 0.92).

None of the condition indices predicted the number of young fledged by successful breeders in either the 4 or 2-year datasets as evidenced by substantial model-uncertainty with the top models having 17% and 9% of the weight, respectively (S2 and S3 Tables). The baseline models are among the top models in both cases.

Scaled mass is the best supported condition index for predicting the number of young produced that survive to independence. The top two models in both the 4 and 2-year datasets are a model with a quadratic term for scaled mass and a model with a linear effect of scaled mass. Combined, these two models have 41% and 70% of the weight in the 4 and 2-year datasets, respectively. The evidence ratios for the top model (scaled mass + scaled mass^2^) are 7.0 and 22.0 against the baseline model, and 2.2 and 1.7 against the linear model of scaled mass for the 4 and 2-year datasets, respectively. In the 4-year dataset, the third best model (PC2 + PC2^2^) is within two AICc units of the top model and has an evidence ratio of 2.8 against the baseline model, and 2.2 against the linear model of PC2.

**Fig 2:**
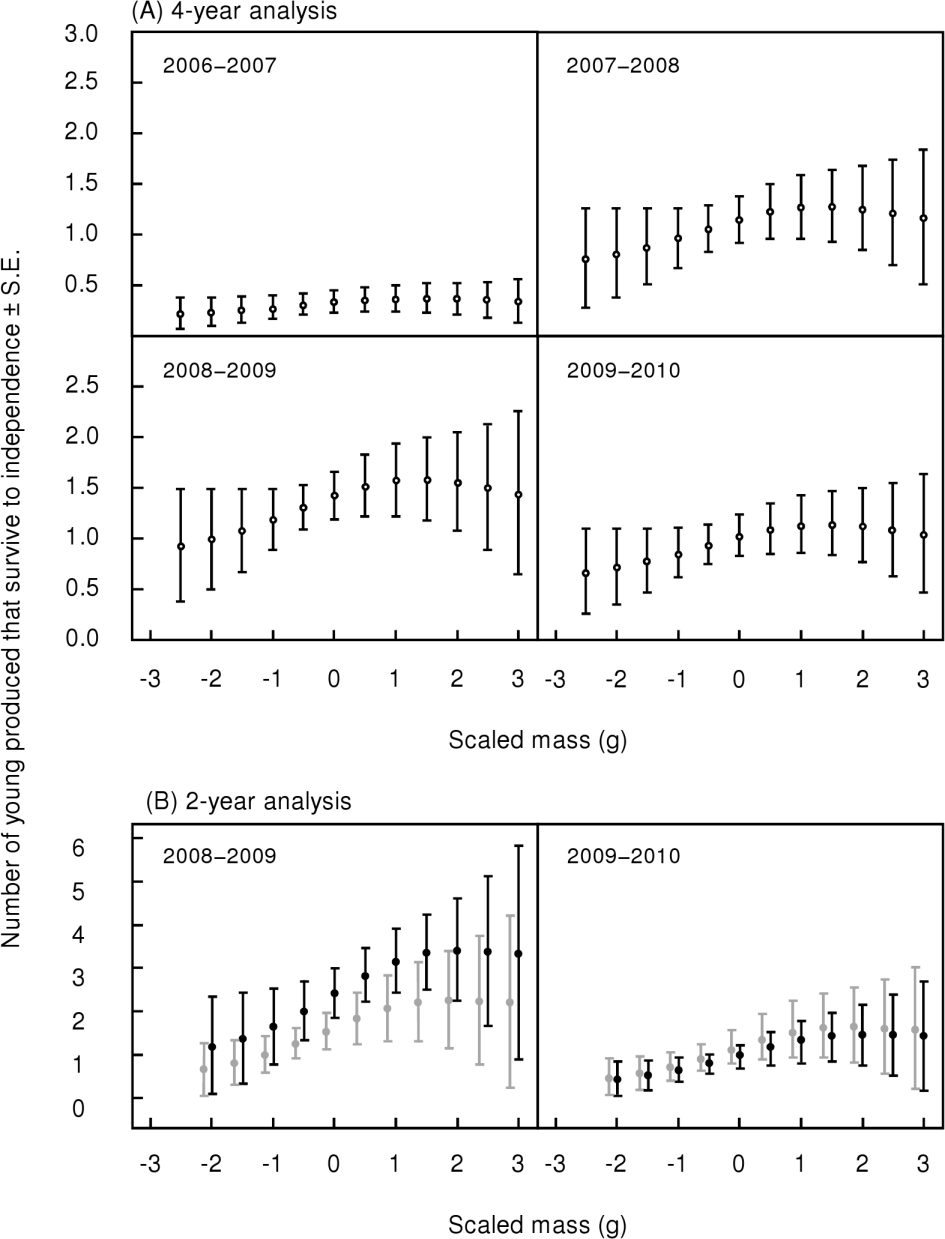
Predictions of the number of young produced that survive to independence by scaled mass. Predictions are model-averaged and reflect the number of young in one breeding season. Scaled mass was centered to have a mean of zero such that the scale of the x-axis is the difference in grams from the mean. Predictions are presented for the 4-year analysis (A) which corresponds to the 2006−2007, 2007−2008, 2008−2009 and 2009−2010 breeding seasons, and for the 2-year analysis (B) which corresponds to the 2008−2009 and 2009−2010 breeding seasons. Individuals of any age are shown with unfilled circles; after-first year breeders in black; and those in their first breeding season in grey. Note that the scale of the y-axis differs between the 4-year (A) and 2-year (B) panels.

Scaled mass had a positive effect on the number of young that survived to independence from low to above average mass, but this effect then plateaued at the highest values of scaled mass (Fig 2). The effect of scaled mass on reproductive success from the 4-year analysis (Fig 2A) is qualitatively similar to that from the 2-year analysis (Fig 2B), but is weaker, exhibits less variation and is not evident in all years. From the 2-year analysis, birds with optimal scaled mass are predicted to have an approximately three-fold increase in reproductive success over birds with low scaled mass: during an average year for reproductive success (2009−2010), an individual at an optimal scaled mass in at least their second-breeding season is predicted to produce 1.5 ± 0.7 young that survive to independence compared to 0.5 ± 0.4 young for an individual with a relatively low scaled mass (Fig 2B). During the year with high population-wide reproductive success (2008−2009), individuals of optimal scaled mass are predicted to produce 3.4 ± 1.2 young compared to 1.2 ± 1.1 young for individuals with low scaled mass (Fig 2B).

Standard error is substantial around some of the model-averaged predictions in Fig 2 due to (a) smaller sample sizes at the extreme high and low ends of the scaled mass axis, (b) variation in the raw data (number of young produced that survive to independence ranged from 0–7 young), and (c) the large proportion of individuals that fledged no young in all years and categories.

### Survival

The model-averaged apparent monthly survival rate was 0.95 (0.94−0.96, 95% CI) from the 4-year dataset, and 0.96 (0.91−0.98) from the 2-year dataset. The model-averaged recapture rate varied monthly from 0.50 (0.32−0.68) to 1 (1−1) and from 0.82 (0.63−0.92) to 1 (1−1) for the 4 and 2-year datasets, respectively. Complete QAICc results are provided in S1 Table.

None of the condition indices predict survival as evidenced by high model uncertainty in all analyses with the top models only having 10−16% of the weight (S1 Table). Fat and PC2 in the 2-year dataset improved model fit over the baseline model but the baseline model was competitive with the top model in this and the 4-year dataset (S1 Table).

## Discussion

We tested the common interpretation of condition indices as proxies for fitness by asking if condition indices predict reproductive success and survival. We found only partial support for this hypothesis because although two condition indices predict annual reproductive success, most condition indices fail to do so and none predict survival. We conclude that condition indices are sometimes, but not always predictors of fitness components, and that a more nuanced interpretation of condition is needed.

### Condition indices and reproductive success

Although none of the condition indices predict the number of young fledged, less model uncertainty combined with higher evidence ratios suggest that some condition indices predict both the probability of fledging at least one young and the number of young produced that survive to independence (in at least some years).

The strongest support for a relationship between a condition index and fitness is that the probability of an adult successfully fledging young varies by PC2. Because nearly half of all breeding *Neochmia phaeton* fail to fledge any young in a given breeding season [17], the probability of fledging young is an important component of fitness. The relationship is strongest in the pre-breeding stage, becoming progressively weaker the closer the individual is to fledging young (Fig 1). Thus condition immediately before the onset of breeding predicts the probability that an individual will successfully fledge young up to five months later. PC2 is positively weighted by all of the condition indices from the 4-year dataset (packed cell volume, hemoglobin, scaled mass, muscle and fat scores) such that one end of the axis represents individuals with high energy reserves and high oxygen-carrying capacity, while the opposite end represents low energy reserves and low oxygen-carrying capacity (generally interpreted as good and poor condition, respectively). Although the axis is a combination of all indices, it is influenced most by muscle score (loading 0.61) and to a lesser extent by fat score (loading 0.48, Table 2). However, adding muscle or fat score alone did not improve model fit over the baseline model (S2 Table), indicating that the multivariate approach of assessing multiple physiological systems concurrently manifested in the PCA provides meaningful information beyond that resulting from examining condition indices individually. For example, having a lot of muscle does not incur fitness benefits, but having a lot of muscle in conjunction with high values for the other indices does. Concurrently maintaining various physiological systems appears to be important for fitness and therefore assessing condition across these systems in a multivariate framework is a useful approach. Why muscle score is an important variable in predicting the probability of fledging young but not in predicting our other measures of reproductive success remains unclear.

Our finding that the average value of PC2 is least adaptive and that the extremes are most optimal was unexpected and the reason for this pattern is not immediately obvious. We attempted to elucidate this pattern by using post-hoc tests to compare individuals in the lower and upper quartiles of PC2, but we found no differences between the groups. This leaves unexplained the pattern that those with low energy reserves and oxygen-carrying capacity are equally as successful at fledging young as those with high energy reserves and oxygen-carrying capacity.

Moderate support from evidence ratios and model weights suggest that individuals that were heavier for their body size produced more independent young than those with average or below average mass for their body size. Some caveats to this conclusion are that (a) considerable model uncertainty exists suggesting that other models have some (although relatively weak) support, (b) evidence ratios for the effect of scaled mass are moderate but not strong, (c) the pattern is only evident in some, but not all years, and (d) data limitations caused wide margins of error in our model-averaged predictions (see Results) and should therefore be interpreted cautiously. Despite these considerations, the evidence indicates that in at least some years, scaled mass has a positive effect on reproductive success, an effect that persists even after averaging the effect across all models including those that do not include scaled mass.

That an individual may increase their annual reproductive success three-fold by optimizing their mass is striking. This pattern suggests that those individuals able to maintain energy reserves are most likely to be able to carry reproduction through to completion. Thus, although individuals with low energy reserves (i.e., low PC2 scores) have the same probability of fledging at least one young as do those with high energy reserves, they are less likely to have their young survive to independence, indicating that this is a less effective strategy for maximizing fitness than that represented by high PC2 scores.

Others have also found that energy reserves are positively related to fecundity, for example among *Chen caerulescens* (snow geese, [27]) and *Somateria mollissima* (common eider, [28,29]). However, these are extreme examples, and not universal even among precocial birds (reviewed by [30]). Here we provide an example of this relationship from a small passerine whose breeding biology clearly differs from that of capital breeders. Passerines are generally income breeders [31] and our findings that heavier individuals have higher reproductive success supports the broad premise of condition indices as proxies for fitness: that individuals with more energy reserves allocate these additional resources toward improving their fitness.

However, additional energy reserves do not always improve reproductive success. Although scaled mass predicted reproductive success in three out of four years in our study, it was uninformative in 2006 − 2007 (Fig 2A). This breeding season had low rainfall as well as unusual timing of rainfall which may be unfavorable for breeding by *Neochmia phaeton*. Among *Branta bernicla* (Brent geese), unfavorable environmental conditions limited the positive effect of scaled mass on reproductive success [32] and a similar mechanism may explain both the especially low reproductive success during 2006 − 2007 in our study and our finding that the effect of scaled mass on reproductive success is inconsistent across years (Fig 2). Elsewhere, environmental conditions, along with parental experience, were more important than mass adjusted for size in explaining an important reproductive trait [33]. Thus, while energy stores predict reproductive success in some contexts, reproduction in other contexts (even within a population) may be constrained by alternative factors such that energy reserves are less influential. This complicates the interpretation of condition indices. Furthermore, mass adjusted for size in other species failed to explain variation in clutch size and number of young fledged [34], and the probability of double-brooding [35] suggesting that in some cases condition indices are simply not relevant. No condition index can function as a reliable, universal proxy of fitness: condition may not be among the important factors affecting fitness in some cases, and where it is, it may be context-dependent. It is therefore challenging to predict under which circumstances condition indices will be meaningful.

Unlike the traditional condition indices that reflect energy reserves, hematological indices did not predict reproductive success among *Neochmia phaeton*. Others have also found that hematological condition indices do not predict fecundity parameters such as the probability of double-brooding [35], number of eggs laid or young fledged [34], and laying date or clutch size [36]. However, this result is not universal: re-nesting birds had higher total plasma protein values than those not re-nesting [7], glycosylated hemoglobin and plasma protein were positively correlated with both clutch size and number of young fledged [37], and mean corpuscular volume (but not packed cell volume) predicted the number of young fledged [6]. Therefore both traditional and hematological indices may sometimes, but not always, be meaningful indicators of reproductive success. Our concern is that there is no clear way to predict which condition indices may be informative, and for which species and under which circumstances.

### Condition indices and survival

Condition indices have no relationship to the survival of *Neochmia phaeton*, as evidenced by high model uncertainty and competitive null models. Others have found that condition indices do predict survival [38] or that they only predict survival during particularly challenging times. For example, mass adjusted for structural body size predicted the survival of *Aphelocoma coerulescens* through a disease epidemic [39] but not otherwise (T. Wilcoxen, personal communication) and fat scores predicted the survival of wintering *Parus major* when they were food limited but not otherwise, and only among subordinate but not dominant classes [40]. Also, stress physiology predicted the survival of marine iguanas during a starvation event [41,42], and although corticosterone is not strictly a condition index it is relevant here because it is involved in the metabolism of energy reserves. During our study, we did not observe such dramatic selective events suggesting that perhaps the environment was not sufficiently challenging for condition to be meaningful for survival. However, our study area has a variable environment with wet season (December—March) rainfall varying from 339 mm to 870 mm during our study, and considerable annual variation in reproductive success exists, suggesting that environmental conditions are not always ideal and that some years may indeed be challenging. Yet condition indices failed to predict survival even during these years.

One reason that condition indices failed to predict survival in our study may be that the indices we measured are not relevant to the survival of our study species. For example, hemoglobin concentration predicted the survival of *Enhydra lutris* (sea otter) while fat reserves did not, and the natural history and physiology of *Enhydra lutris* may explain this difference: fat is quickly utilized due to the otters’ high metabolic rate, and as diving foragers, it is oxygen-carrying capacity that determines their ability to acquire food [8]. Therefore, it is hemoglobin concentration and not fat that is the more meaningful condition index for this species [8]. It is not uncommon that some condition indices predict survival while others in the same study do not [8,10,39,43]. Therefore, condition indices are not broadly applicable and should be selected for use based on their relevance to the study organism. For example, fat reserves can generally be interpreted as being beneficial for survival among migrating birds and species that face unpredictable thermal challenges, but it is unclear whether fat scores are relevant among sedentary, tropical passerines such as *Neochmia phaeton*, for which the costs and benefits of fat reserves and their associated trade-offs are poorly understood. Predicting which condition indices are most relevant may be easiest for species that have extreme physiological demands.

Alternatively, condition indices in our study may have failed to indicate survival of *Neochmia phaeton* because they were sampled at the wrong time of year. To predict survival condition indices likely need to be sampled prior to, or during, the challenge that contributes most to mortality. We do not know the primary sources of mortality for adults in our study population. However, *Neochmia phaeton* occupies a seasonal, tropical environment with distinct dry and wet seasons, and we suspect that the most food-limiting time is the end of the dry season when grass seeds are most depleted [44]. If we had measured condition at this time, rather than during the wet season breeding period when *Neochmia phaeton* are less food-stressed, condition indices may have predicted survival (but perhaps not subsequent reproductive success). We encourage researchers to consider the relevance of both the condition indices and the timing of sampling in employing these indices as predictors of fitness.

## Conclusions

Although traditional condition indices predict reproductive success among *Neochmia phaeton*, most of the indices that we measured fail to do so, and none predict survival. These results and other literature indicate that condition indices are only sometimes potentially meaningful proxies for fitness. We therefore question the ubiquitous interpretation of condition indices as proxies for fitness. How then, should condition indices be interpreted? First, as exemplified by our result relating PC2 to reproductive success, indices may be more informative when integrated via a multivariate approach than when assessed individually. Second, where we found that condition indices predicted a component of fitness, its quadratic effect was always better supported than a linear effect of that index. We therefore echo the suggestion of others that more is not necessarily better and that the relationship between a condition index and fitness is not necessarily linear (as in sexual selection theory; reviewed by [4,11,45]).

The wide discrepancy in whether condition indices predict fitness, and if so, which indices in particular are meaningful, suggests that not all indices are relevant to all species or contexts [32,45]. Researchers should consider which physiological aspects of condition would be most informative in their study system, select condition indices based on this justification, and validate their choice of indices. This complicates the use of condition indices because one of their great benefits is that they are easier to sample in many species than is fitness and for this reason are used as its surrogate. Furthermore, as we saw with scaled mass in our study, if a condition index is found to predict a component of fitness, it may only do so during some years and not others, demonstrating that the condition-fitness relationship is not broadly applicable across all contexts. This point was articulated by Lailvaux and Kasumovic [11] who argued that measuring individual quality is context-dependent because a given resource allocation strategy may be adaptive under some contexts but maladaptive under others, and there may be more than one strategy that maximizes fitness over a lifetime. Understanding lifetime resource allocation decisions and their associated trade-offs and relationships to fitness will offer more insights than snapshots of resource allocation (e.g., condition indices).

The interpretation of condition indices as proxies for individual quality and fitness must be refined. We previously found that condition indices are only repeatable within individuals across short, but not long time periods [12] suggesting that condition indices do not indicate inherent individual quality. In this study, we found that condition indices are not reliable proxies for fitness. Together, these two studies suggest that rather than being interpreted as indicators of individual quality or of having long-term consequences for fitness, condition indices instead should be considered as possible indicators of current and short-term success. Condition indices at a single point in time indicate how well an individual is currently acquiring and assimilating resources and, because we previously found condition indices to be repeatable over several months, how well they will likely be faring several months into the future [12]. Although high values of traditional condition indices indicate that the individual currently has relatively more resources, those resources may not necessarily result in fitness benefits. The other indices, such as hematological parameters, provide information about specific systems such as the oxygen carrying capacity of blood. Although these condition indices provide insights into an individual’s current physiological state, they are not proxies for individual quality or fitness. A single snapshot of condition may not be as meaningful as the more complex and long-term physiological processes that influence fitness. For example, individual quality may be better understood through the resource allocation decisions made over a lifetime [11], the individual’s mechanism for regulating their condition [46,47] or the individual’s capacity to maintain optimal functionality of essential cellular processes [4].

## Supporting Information

S1 Table QAICc table of results for the survival analyses of the 4 and 2-year datasets. Included are all of the models from the candidate model set where ϕ = apparent monthly adult survival, ρ = recapture probability, *t* = time dependence, PCV = packed cell volume, Hb = hemoglobin concentration, SMI = scaled mass index, Muscle = muscle score, Fat = fat score, HL = heterophil to lymphocyte ratio, TPP = total plasma protein, Time = time of day, and PCs = principal components. See Methods for an explanation of the covariates including Sex, Stage, Age, Time of Day, and Year. The baseline model is in bold. Also included are the number of parameters in the model (k), the quasi-Akaike's Information Criterion corrected for small sample size (QAICc), the difference in QAICc of a model from that of the top model (ΔQAICc), the model weight (W), and its deviance (Qdev).
(DOCX)

S2 Table AICc table for results for the annual reproductive success analyses of the 4-year datasets. We asked three questions of each dataset for a total of six analyses. Included are all of the models from the candidate model set. PCV = packed cell volume, Hb = hemoglobin concentration, SMI = scaled mass index, Muscle = muscle score, Fat = fat score, HL = heterophil to lymphocyte ratio, TPP = total plasma protein, Time = time of day, and PCs = principal components. See Methods for an explanation of the covariates including Sex, Stage, Age, Time, and Year. The baseline model is in bold. Also included are the number of parameters in the model (k), the Akaike's Information Criterion corrected for small sample size (AICc), the difference in AICc of a model from that of the top model (ΔAICc), the model weight (W), and the log-likelihood of each model (LL). An asterisk (*) indicates that the model failed to converge.
(DOCX)

S3 Table AICc table for results for the annual reproductive success analyses of the 2-year datasets. We asked three questions of each dataset for a total of six analyses. Included are all of the models from the candidate model set. PCV = packed cell volume, Hb = hemoglobin concentration, SMI = scaled mass index, Muscle = muscle score, Fat = fat score, HL = heterophil to lymphocyte ratio, TPP = total plasma protein, Time = time of day, and PCs = principal components. See Methods for an explanation of the covariates including Sex, Stage, Age, Time, and Year. The baseline model is in bold. Also included are the number of parameters in the model (k), the Akaike's Information Criterion corrected for small sample size (AICc), the difference in AICc of a model from that of the top model (ΔAICc), the model weight (W), and the log-likelihood of each model (LL).
(DOCX)
