# Supplementary material for: Body Condition Indices Predict Reproductive Success but Not Survival in a Sedentary, Tropical Bird
Source: PLoS One. 2015 Aug 25;10(8):e0136582. doi: 10.1371/journal.pone.0136582 (PMC4549336; doi:10.1371/journal.pone.0136582)
Supplement: S1 Table — Included are all of the models from the candidate model set where ϕ = apparent monthly adult survival, ρ = recapture probability, t = time dependence, PCV = packed cell volume, Hb = hemoglobin concentration, SMI = scaled mass index, Muscle = muscle score, Fat = fat score, HL = heterophil to lymphocyte ratio, TPP = total plasma protein, Time = time of day, and PCs = principal components. See Methods for an explanation of the covariates including Sex, Stage, Age, Time of Day, and Year. The baseline model is in bold. Also included are the number of parameters in the model (k), the quasi-Akaike's Information Criterion corrected for small sample size (QAICc), the difference in QAICc of a model from that of the top model (ΔQAICc), the model weight (W), and its deviance (Qdev). (DOCX) [file pone.0136582.s001.docx]

**Table S1.** QAICc table of results for the survival analyses of the 4 and 2-year datasets among *Neochmia phaeton*. Included are all of the models from the candidate model set where φ = apparent monthly adult survival, ρ = recapture probability, *t* = time dependence, PCV = packed cell volume, Hb = hemoglobin concentration, SMI = scaled mass index, Muscle = muscle score, Fat = fat score, HL = heterophil to lymphocyte ratio, TPP = total plasma protein, Time = time of day, and PCs = principal components. See Methods for an explanation of the covariates including Sex, Stage, Age, Time of Day, and Year. The baseline model is in bold. Also included are the number of parameters in the model (k), the quasi-Akiake's Information Criterion corrected for small sample size (QAICc), the difference in QAICc of a model from that of the top model (ΔQAICc), the model weight (W), and its deviance (Qdev).

| Dataset | Model | k | QAICc | ΔQAICc | W | Qdev |
| --- | --- | --- | --- | --- | --- | --- |
| 4-year | φ(.) ρ(*t*+Year+Sex) | 25 | 1446.30 | 0.00 | 0.10 | 1395.30 |
|  | **φ(Sex+Age) ρ(*t*+Year+Sex+Stage)** | 33 | 1446.52 | 0.22 | 0.09 | 1378.78 |
|  | φ(Age) ρ(*t*+Year+Sex+Stage) | 32 | 1446.69 | 0.38 | 0.08 | 1381.05 |
|  | φ(Sex+Age+PCV) ρ(*t*+Year+Sex+Stage) | 34 | 1446.75 | 0.44 | 0.08 | 1376.90 |
|  | φ(Sex) ρ(*t*+Year+Sex+Stage) | 31 | 1446.78 | 0.48 | 0.08 | 1383.24 |
|  | φ(.) ρ(*t*+Year+Sex+Stage) | 30 | 1447.26 | 0.95 | 0.06 | 1385.81 |
|  | φ(Sex+Age+PCV+PCV^2^) ρ(*t*+Year+Sex+Stage) | 35 | 1447.53 | 1.23 | 0.05 | 1375.57 |
|  | φ(Sex+Age+Hb+Hb^2^) ρ(*t*+Year+Sex+Stage) | 35 | 1448.18 | 1.88 | 0.04 | 1376.22 |
|  | φ(Sex+Age+PC2) ρ(*t*+Year+Sex+Stage) | 34 | 1448.29 | 1.99 | 0.04 | 1378.44 |
|  | φ(Sex+Age+Hb) ρ(*t*+Year+Sex+Stage) | 34 | 1448.41 | 2.11 | 0.03 | 1378.56 |
|  | φ(Sex+Age+Fat) ρ(*t*+Year+Sex+Stage) | 34 | 1448.51 | 2.20 | 0.03 | 1378.66 |
|  | φ(Sex+Age+SMI) ρ(*t*+Year+Sex+Stage) | 34 | 1448.55 | 2.24 | 0.03 | 1378.70 |
|  | φ(Sex+Age+PC1) ρ(*t*+Year+Sex+Stage) | 34 | 1448.58 | 2.28 | 0.03 | 1378.73 |
|  | φ(Sex+Age+Muscle) ρ(*t*+Year+Sex+Stage) | 34 | 1448.58 | 2.28 | 0.03 | 1378.73 |
|  | φ(Sex+Age+Muscle+Year) ρ(*t*+Year+Sex+Stage) | 37 | 1448.85 | 2.55 | 0.03 | 1372.66 |
|  | φ(Sex+Age+PC1+PC1^2^) ρ(*t*+Year+Sex+Stage) | 35 | 1449.10 | 2.80 | 0.02 | 1377.14 |
|  | φ(Sex+Age+PC2+Year) ρ(*t*+Year+Sex+Stage) | 37 | 1449.15 | 2.85 | 0.02 | 1372.96 |
|  | φ(Sex+Age+Muscle+Muscle^2^) ρ(*t*+Year+Sex+Stage) | 35 | 1449.18 | 2.87 | 0.02 | 1377.22 |
|  | φ(.) ρ(*t*+Year+Sex+Age) | 27 | 1449.37 | 3.07 | 0.02 | 1394.20 |
|  | φ(.) ρ(*t*+Year+Sex+Stage+Age) | 32 | 1449.70 | 3.40 | 0.02 | 1384.06 |
|  | φ(Sex+Age+PC2+PC2^2^) ρ(*t*+Year+Sex+Stage) | 35 | 1449.97 | 3.67 | 0.02 | 1378.01 |
|  | φ(Sex+Age+Fat+Fat^2^) ρ(*t*+Year+Sex+Stage) | 35 | 1450.39 | 4.08 | 0.01 | 1378.43 |
|  | φ(Sex+Age+PC2+PC2^2^+Year) ρ(*t*+Year+Sex+Stage) | 38 | 1450.41 | 4.11 | 0.01 | 1372.10 |
|  | φ(Sex+Age+SMI+SMI^2^) ρ(*t*+Year+Sex+Stage) | 35 | 1450.57 | 4.27 | 0.01 | 1378.61 |
|  | φ(Sex+Age+Muscle+Muscle^2^+Year) ρ(*t*+Year+Sex+Stage) | 38 | 1450.68 | 4.38 | 0.01 | 1372.37 |
|  | φ(.) ρ(t+Year+Sex+Age+(Sex × Age)) | 29 | 1451.68 | 5.37 | 0.01 | 1392.33 |
|  | φ(.) ρ(t+Year+Sex+Age+(Sex × Age)+Stage) | 34 | 1451.87 | 5.57 | 0.01 | 1382.03 |
|  | φ(Sex+Age+Fat+Stage+Year) ρ(*t*+Year+Sex+Stage) | 42 | 1452.97 | 6.67 | 0.00 | 1366.15 |
|  | φ(Sex+Age+Fat+Fat^2^+Stage+Year) ρ(*t*+Year+Sex+Stage) | 43 | 1454.04 | 7.73 | 0.00 | 1365.07 |
|  | φ(Sex+Age+PCV+Stage+(Sex × Stage)) ρ(t+Year+Sex+Stage) | 44 | 1459.11 | 12.80 | 0.00 | 1368.00 |
|  | φ(Sex+Age+PCV+PCV^2^+Stage+(Sex × Stage)) ρ(t+Year+Sex+Stage) | 45 | 1461.14 | 14.84 | 0.00 | 1367.90 |
|  | φ(Sex+Age+SMI+Stage+(Sex × Stage)+Time) ρ(t+Year+Sex+Stage) | 45 | 1461.35 | 15.05 | 0.00 | 1368.11 |
|  | φ(Sex+Age+Hb+Stage+(Sex × Stage)+Year) ρ(t+Year+Sex+Stage) | 47 | 1461.51 | 15.21 | 0.00 | 1363.97 |
|  | φ(Sex+Age+PC1+Stage+(Sex × Stage)+Year) ρ(t+Year+Sex+Stage) | 47 | 1461.53 | 15.23 | 0.00 | 1363.99 |
|  | φ(Sex+Age+SMI+SMI^2^+Stage+(Sex × Stage)+Time) ρ(t+Year+Sex+Stage) | 46 | 1463.17 | 16.87 | 0.00 | 1367.78 |
|  | φ(Sex+Age+Hb+Hb^2^+Stage+(Sex × Stage)+Year) ρ(t+Year+Sex+Stage) | 48 | 1463.64 | 17.34 | 0.00 | 1363.94 |
|  | φ(Sex+Age+PC1+PC1^2^+Stage+(Sex × Stage)+Year) ρ(t+Year+Sex+Stage) | 48 | 1463.66 | 17.36 | 0.00 | 1363.97 |
|  | φ(.) ρ(t+Year+Sex+Stage+Age+(Stage × Age)+Sex) | 42 | 1465.59 | 19.29 | 0.00 | 1378.77 |
|  | φ(.) ρ(t+Year+Sex+Stage+Age+(Stage × Age)+Sex+(Sex × Age)) | 44 | 1468.62 | 22.32 | 0.00 | 1377.52 |
|  | φ(.) ρ(*t*+Year+Stage) | 29 | 1468.67 | 22.37 | 0.00 | 1409.32 |
|  | φ(.) ρ(*t*+Year+Stage+Age) | 31 | 1470.70 | 24.40 | 0.00 | 1407.17 |
|  | φ(.) ρ(*t*+Year) | 24 | 1476.42 | 30.12 | 0.00 | 1427.50 |
|  | φ(.) ρ(*t*+Year+Age) | 26 | 1479.01 | 32.70 | 0.00 | 1425.92 |
|  | φ(Year) ρ(*t*+Year) | 27 | 1479.24 | 32.94 | 0.00 | 1424.08 |
|  | φ(t) ρ(*t*+Year) | 42 | 1484.46 | 38.16 | 0.00 | 1397.64 |
|  | φ(.) ρ(*t*+Year+Sex+Stage+Age+(Stage × Age)) | 41 | 1485.85 | 39.55 | 0.00 | 1401.16 |
|  | φ(*t*+Year) ρ(*t*+Year) | 45 | 1490.07 | 43.77 | 0.00 | 1396.82 |
|  | φ(*t*+Year+(*t* × Year)) ρ(*t*+Year) | 102 | 1575.94 | 129.63 | 0.00 | 1354.70 |
|  | φ(*t*+Year+(*t* × Year)) ρ(*t*) | 99 | 1588.99 | 142.69 | 0.00 | 1374.79 |
|  | φ(*t*+Year+(*t* × Year)) ρ(Year) | 84 | 1615.02 | 168.72 | 0.00 | 1435.48 |
|  | φ(*t*+Year+(*t* × Year)) ρ(.) | 81 | 1649.92 | 203.62 | 0.00 | 1477.21 |
|  | φ(*t*+Year+(*t* × Year)) ρ(*t*+Year+(*t* × Year)) | 159 | 1655.79 | 209.49 | 0.00 | 1294.01 |
|  |  |  |  |  |  |  |
| 2-year | φ(Year+Sex+Fat+Fat^2^) ρ(*t*+Sex) | 13 | 291.74 | 0.00 | 0.16 | 264.63 |
|  | φ(Year+Sex+PC2+PC2^2^) ρ(*t*+Sex) | 13 | 292.83 | 1.09 | 0.09 | 265.72 |
|  | **φ(Year+Sex) ρ(*t*+Sex)** | 11 | 293.18 | 1.44 | 0.08 | 270.38 |
|  | φ(Year+Sex+Muscle) ρ(*t*+Sex) | 12 | 293.41 | 1.67 | 0.07 | 268.46 |
|  | φ(Year+Sex+SMI) ρ(*t*+Sex) | 12 | 293.81 | 2.07 | 0.06 | 268.86 |
|  | φ(Year+Sex+PC3) ρ(*t*+Sex) | 12 | 293.99 | 2.25 | 0.05 | 269.04 |
|  | φ(Year+Sex+PC2) ρ(*t*+Sex) | 12 | 294.01 | 2.27 | 0.05 | 269.05 |
|  | φ(Year+Sex+SMI+SMI^2^) ρ(*t*+Sex) | 13 | 294.13 | 2.39 | 0.05 | 267.02 |
|  | φ(Year+Sex+HL) ρ(*t*+Sex) | 12 | 294.41 | 2.67 | 0.04 | 269.46 |
|  | φ(Year+Sex+Hb) ρ(*t*+Sex) | 12 | 294.59 | 2.85 | 0.04 | 269.64 |
|  | φ(Year+Sex+Muscle+Muscle^2^) ρ(*t*+Sex) | 13 | 294.84 | 3.10 | 0.03 | 267.73 |
|  | φ(Year+Sex+Fat) ρ(*t*+Sex) | 12 | 295.00 | 3.26 | 0.03 | 270.05 |
|  | φ(Year+Sex+TPP) ρ(*t*+Sex) | 12 | 295.23 | 3.49 | 0.03 | 270.28 |
|  | φ(Year+Sex+PC1) ρ(*t*+Sex) | 12 | 295.32 | 3.58 | 0.03 | 270.37 |
|  | φ(Year+Sex+PCV) ρ(*t*+Sex) | 12 | 295.33 | 3.59 | 0.03 | 270.38 |
|  | φ(Year+Sex+Age) ρ(*t*+Sex) | 13 | 295.42 | 3.69 | 0.03 | 268.31 |
|  | φ(Year+Sex+PC3+PC3^2^) ρ(*t*+Sex) | 13 | 295.83 | 4.09 | 0.02 | 268.72 |
|  | φ(Year+Sex+HL+HL^2^) ρ(*t*+Sex) | 13 | 296.00 | 4.26 | 0.02 | 268.88 |
|  | φ(Year) ρ(*t*+Sex) | 10 | 296.34 | 4.60 | 0.02 | 275.67 |
|  | φ(Year+Sex+Hb+Hb^2^) ρ(*t*+Sex) | 13 | 296.75 | 5.01 | 0.01 | 269.64 |
|  | φ(Year+Sex+PC1+PC1^2^) ρ(*t*+Sex) | 13 | 296.92 | 5.18 | 0.01 | 269.80 |
|  | φ(Year+Sex+PCV+PCV^2^) ρ(*t*+Sex) | 13 | 297.31 | 5.57 | 0.01 | 270.19 |
|  | φ(Year+Sex+TPP+TPP^2^) ρ(*t*+Sex) | 13 | 297.38 | 5.64 | 0.01 | 270.26 |
|  | φ(Year+Age) ρ(*t*+Sex) | 12 | 298.05 | 6.31 | 0.01 | 273.10 |
|  | φ(Year+Sex+PC3+Stage) ρ(*t*+Sex) | 17 | 298.62 | 6.89 | 0.01 | 262.73 |
|  | φ(.) ρ(*t*) | 8 | 298.86 | 7.12 | 0.00 | 282.43 |
|  | φ(Year+Sex+Fat+Fat^2^+Stage) ρ(*t*+Sex) | 18 | 299.01 | 7.27 | 0.00 | 260.88 |
|  | φ(Year+Sex+Age+(Sex × Age)) ρ(*t*+Sex) | 15 | 299.59 | 7.85 | 0.00 | 268.11 |
|  | φ(Year) ρ(*t*+Sex+Age) | 12 | 299.65 | 7.91 | 0.00 | 274.70 |
|  | φ(Year) ρ(*t*+Sex+Age+(Sex × Age)) | 14 | 300.14 | 8.40 | 0.00 | 270.85 |
|  | φ(Year) ρ(*t*+Age) | 11 | 300.37 | 8.64 | 0.00 | 277.57 |
|  | φ(Year) ρ(*t*) | 9 | 300.44 | 8.70 | 0.00 | 281.90 |
|  | φ(Year+Sex+PC3+PC3^2^+Stage) ρ(*t*+Sex) | 18 | 300.83 | 9.09 | 0.00 | 262.71 |
|  | φ(Year+Sex+Fat+Stage) ρ(*t*+Sex) | 17 | 300.85 | 9.11 | 0.00 | 264.95 |
|  | φ(*t*) ρ(*t*) | 13 | 305.17 | 13.44 | 0.00 | 278.06 |
|  | φ(*t*+Year) ρ(*t*) | 14 | 305.46 | 13.72 | 0.00 | 276.17 |
|  | φ(Year) ρ(*t*+Sex+Stage) | 15 | 305.77 | 14.03 | 0.00 | 274.29 |
|  | φ(Year) ρ(*t*+Stage) | 14 | 308.67 | 16.93 | 0.00 | 279.38 |
|  | φ(Year+Sex+SMI+SMI^2^+Stage+(Sex × Stage)+Time) ρ(*t*+Sex) | 23 | 309.32 | 17.58 | 0.00 | 259.84 |
|  | φ(Year) ρ(*t*+Sex+Stage+Age) | 17 | 309.45 | 17.71 | 0.00 | 273.56 |
|  | φ(Year+Sex+SMI+Stage+(Sex × Stage)+Time) ρ(*t*+Sex) | 22 | 309.65 | 17.92 | 0.00 | 262.47 |
|  | φ(Year) ρ(*t*+Stage+Age) | 16 | 309.67 | 17.93 | 0.00 | 275.99 |
|  | φ(Year+Sex+Hb+Stage+(Sex × Stage)) ρ(*t*+Sex) | 22 | 310.05 | 18.31 | 0.00 | 262.87 |
|  | φ(Year) ρ(*t*+Sex+Age+(Sex × Age)+Stage) | 19 | 310.43 | 18.69 | 0.00 | 270.06 |
|  | φ(Year+Sex+PC1+Stage+(Sex × Stage)) ρ(*t*+Sex) | 22 | 311.06 | 19.32 | 0.00 | 263.87 |
|  | φ(Year+Sex+PCV+Stage+(Sex × Stage)) ρ(*t*+Sex) | 22 | 311.06 | 19.32 | 0.00 | 263.88 |
|  | φ(Year+Sex+TPP+Stage+(Sex × Stage)) ρ(*t*+Sex) | 22 | 311.06 | 19.32 | 0.00 | 263.88 |
|  | φ(Year) ρ(*t*+Sex+Stage+(Sex × Stage)) | 20 | 311.07 | 19.33 | 0.00 | 268.44 |
|  | φ(Year+Sex+Hb+Hb^2^+Stage+(Sex × Stage)) ρ(*t*+Sex) | 23 | 312.01 | 20.27 | 0.00 | 262.53 |
|  | φ(Year+Sex+PCV+PCV^2^+Stage+(Sex × Stage)) ρ(*t*+Sex) | 23 | 312.12 | 20.38 | 0.00 | 262.64 |
|  | φ(Year+Sex+PC1+PC1^2^+Stage+(Sex × Stage)) ρ(*t*+Sex) | 23 | 313.22 | 21.48 | 0.00 | 263.73 |
|  | φ(Year+Sex+TPP+TPP^2^+Stage+(Sex × Stage)) ρ(*t*+Sex) | 23 | 313.25 | 21.52 | 0.00 | 263.77 |
|  | φ(Year) ρ(*t*+Sex+Stage+(Sex × Stage)+Age) | 22 | 315.56 | 23.82 | 0.00 | 268.38 |
|  | φ(*t*+Year+(*t* × Year)) ρ(*t*) | 20 | 316.04 | 24.30 | 0.00 | 273.41 |
|  | φ(Year) ρ(*t*+Sex+Stage+(Sex × Stage)+Age+(Sex × Age)) | 24 | 316.20 | 24.46 | 0.00 | 264.40 |
|  | φ(*t*+Year+(*t* × Year)) ρ(*t*+Year) | 21 | 318.10 | 26.36 | 0.00 | 273.20 |
|  | φ(Year) ρ(*t*+Stage+Age+(Stage × Age)+Sex) | 27 | 321.39 | 29.65 | 0.00 | 262.56 |
|  | φ(Year) ρ(*t*+Stage+Age+(Stage × Age)+Sex+(Sex × Age)) | 29 | 322.11 | 30.37 | 0.00 | 258.51 |
|  | φ(Year) ρ(*t*+Stage+Age+(Stage × Age)) | 26 | 322.15 | 30.41 | 0.00 | 265.67 |
|  | φ(Year) ρ(*t*+Sex+Stage+(Sex × Stage)+Age+(Sex × Age)+(Stage × Age)) | 34 | 323.77 | 32.03 | 0.00 | 248.00 |
|  | φ(*t*+Year+(*t* × Year)) ρ(Year) | 16 | 327.67 | 35.93 | 0.00 | 293.99 |
|  | φ(*t*+Year+(*t* × Year)) ρ(.) | 15 | 328.41 | 36.67 | 0.00 | 296.94 |
|  | φ(Year) ρ(*t*+Sex+Stage+(Sex × Stage)+Age+(Stage × Age)) | 32 | 328.73 | 36.99 | 0.00 | 257.87 |
|  | φ(*t*+Year+(*t* × Year)) ρ(*t*+Year+(*t* × Year)) | 27 | 329.06 | 37.32 | 0.00 | 270.23 |
