# Supplementary material for: Body Condition Indices Predict Reproductive Success but Not Survival in a Sedentary, Tropical Bird
Source: PLoS One. 2015 Aug 25;10(8):e0136582. doi: 10.1371/journal.pone.0136582 (PMC4549336; doi:10.1371/journal.pone.0136582)
Supplement: S2 Table — We asked three questions of each dataset for a total of six analyses. Included are all of the models from the candidate model set. PCV = packed cell volume, Hb = hemoglobin concentration, SMI = scaled mass index, Muscle = muscle score, Fat = fat score, HL = heterophil to lymphocyte ratio, TPP = total plasma protein, Time = time of day, and PCs = principal components. See Methods for an explanation of the covariates including Sex, Stage, Age, Time, and Year. The baseline model is in bold. Also included are the number of parameters in the model (k), the Akaike's Information Criterion corrected for small sample size (AICc), the difference in AICc of a model from that of the top model (ΔAICc), the model weight (W), and the log-likelihood of each model (LL). An asterisk (*) indicates that the model failed to converge. (DOCX) [file pone.0136582.s002.docx]

**Table S2.** AICc table of results for the annual reproductive success analyses of the 4-year datasets among *Neochmia phaeton*. We asked three questions of each dataset for a total of six analyses. Included are all of the models from the candidate model set. PCV = packed cell volume, Hb = hemoglobin concentration, SMI = scaled mass index, Muscle = muscle score, Fat = fat score, HL = heterophil to lymphocyte ratio, TPP = total plasma protein, Time = time of day, and PCs = principal components. See Methods for an explanation of the covariates including Sex, Stage, Age, Time, and Year. The baseline model is in bold. Also included are the number of parameters in the model (k), the Akiake's Information Criterion corrected for small sample size (AICc), the difference in AICc of a model from that of the top model (ΔAICc), the model weight (W), and the log-likelihood of each model (LL). An asterisk (*) indicates that the model failed to converge.

| Response variable | | | Model | | | | | k | AICc | | | ΔAICc | | W | | LL |  |
| --- | --- | --- | --- | --- | --- | --- | --- | --- | --- | --- | --- | --- | --- | --- | --- | --- | --- |
| Fledge or not fledge | | | | | Stage+PC2+PC2^2^ | | | 9 | | 435.53 | 0.00 | | 0.66 | | -208.48 | | |
|  | | | | | Stage+PC2+PC2^2^+Year | | | 12 | | 440.77 | 5.24 | | 0.05 | | -207.89 | | |
|  | | | | | **Stage** | | | 7 | | 440.81 | 5.28 | | 0.05 | | -213.23 | | |
|  | | | | | Stage+Muscle | | | 8 | | 441.55 | 6.03 | | 0.03 | | -212.55 | | |
|  | | | | | Stage+SMI | | | 8 | | 441.98 | 6.45 | | 0.03 | | -212.76 | | |
|  | | | | | Stage+PCV | | | 8 | | 442.15 | 6.63 | | 0.02 | | -212.85 | | |
|  | | | | | Stage+Muscle+Muscle^2^ | | | 9 | | 442.30 | 6.78 | | 0.02 | | -211.87 | | |
|  | | | | | Stage+Fat | | | 8 | | 442.40 | 6.87 | | 0.02 | | -212.98 | | |
|  | | | | | Stage+Hb | | | 8 | | 442.41 | 6.89 | | 0.02 | | -212.98 | | |
|  | | | | | Stage+PC2 | | | 8 | | 442.71 | 7.18 | | 0.02 | | -213.13 | | |
|  | | | | | Stage+PC1 | | | 8 | | 442.72 | 7.20 | | 0.02 | | -213.14 | | |
|  | | | | | Stage+Fat+Fat^2^ | | | 9 | | 443.12 | 7.59 | | 0.01 | | -212.28 | | |
|  | | | | | Stage+SMI+SMI^2^ | | | 9 | | 443.80 | 8.27 | | 0.01 | | -212.62 | | |
|  | | | | | Stage+PCV+PCV^2^ | | | 9 | | 444.26 | 8.73 | | 0.01 | | -212.85 | | |
|  | | | | | Stage+Hb+Hb^2^ | | | 9 | | 444.52 | 8.99 | | 0.01 | | -212.98 | | |
|  | | | | | Stage+PC1+PC1^2^ | | | 9 | | 444.84 | 9.31 | | 0.01 | | -213.14 | | |
|  | | | | | Stage+Year | | | 10 | | 445.25 | 9.73 | | 0.01 | | -212.28 | | |
|  | | | | | Stage+Muscle+Year | | | 11 | | 445.61 | 10.09 | | 0.00 | | -211.39 | | |
|  | | | | | Stage+SMI+Sex+(Sex × Stage) | | | 15 | | 446.63 | 11.10 | | 0.00 | | -207.55 | | |
|  | | | | | Stage+Muscle+Muscle^2^+Year | | | 12 | | 446.90 | 11.37 | | 0.00 | | -210.96 | | |
|  | | | | | Stage+PC2+Year | | | 11 | | 447.15 | 11.62 | | 0.00 | | -212.16 | | |
|  | | | | | Stage+PCV+Sex+(Sex × Stage) | | | 14 | | 447.53 | 12.00 | | 0.00 | | -209.10 | | |
|  | | | | | Stage+SMI+SMI^2^+Sex(Sex × Stage) | | | 16 | | 448.25 | 12.73 | | 0.00 | | -207.26 | | |
|  | | | | | Stage+Fat+Sex | | | 12 | | 448.76 | 13.23 | | 0.00 | | -211.89 | | |
|  | | | | | Stage+PCV+PCV^2^+Sex(Sex × Stage) | | | 15 | | 449.60 | 14.07 | | 0.00 | | -209.04 | | |
|  | | | | | Stage+Fat+Fat^2^+Sex | | | 13 | | 450.04 | 14.51 | | 0.00 | | -211.45 | | |
|  | | | | | Stage+Hb+Sex+(Sex × Stage)+Year | | | 17 | | 452.25 | 16.72 | | 0.00 | | -208.15 | | |
|  | | | | | Stage+PC1+Sex+(Sex × Stage) | | | 17 | | 452.63 | 17.10 | | 0.00 | | -208.34 | | |
|  | | | | | Stage+Hb+Hb^2^+Sex+(Sex × Stage)+Year | | | 18 | | 454.48 | 18.95 | | 0.00 | | -208.15 | | |
|  | | | | | Stage+PC1+PC1^2^+Sex(Sex × Stage) | | | 18 | | 454.82 | 19.29 | | 0.00 | | -208.32 | | |
|  | | | | | Null | | | 2 | | 461.17 | 25.64 | | 0.00 | | -228.56 | | |
|  | | | | | Year | | | 5 | | 462.37 | 26.84 | | 0.00 | | -226.09 | | |
|  | | | | |  | | |  | |  |  | |  | |  | | |
| Number fledged | | | | | **Null** | | | 2 | | 170.15 | 0.00 | | 0.17 | | -83.04 | | |
|  | | | | | PC2+PC2^2^ | | | 4 | | 171.60 | 1.45 | | 0.08 | | -81.69 | | |
|  | | | | | Hb+Hb^2^ | | | 4 | | 171.90 | 1.74 | | 0.07 | | -81.84 | | |
|  | | | | | PCV | | | 3 | | 172.12 | 1.97 | | 0.06 | | -82.99 | | |
|  | | | | | SMI | | | 3 | | 172.19 | 2.03 | | 0.06 | | -83.03 | | |
|  | | | | | Hb | | | 3 | | 172.19 | 2.03 | | 0.06 | | -83.03 | | |
|  | | | | | PC2* | | | 3 | | 172.19 | 2.04 | | 0.06 | | -83.03 | | |
|  | | | | | Muscle* | | | 3 | | 172.20 | 2.05 | | 0.06 | | -83.03 | | |
|  | | | | | Fat | | | 3 | | 172.22 | 2.07 | | 0.06 | | -83.04 | | |
|  | | | | | PC1 | | | 3 | | 172.22 | 2.07 | | 0.06 | | -83.04 | | |
|  | | | | | PC1+PC1^2^* | | | 4 | | 172.77 | 2.61 | | 0.05 | | -82.27 | | |
|  | | | | | Year | | | 5 | | 173.06 | 2.91 | | 0.04 | | -81.36 | | |
|  | | | | | Muscle+Muscle^2^ | | | 4 | | 173.80 | 3.64 | | 0.03 | | -82.79 | | |
|  | | | | | Fat+Fat^2^ | | | 4 | | 174.03 | 3.87 | | 0.02 | | -82.90 | | |
|  | | | | | PCV+PCV^2^ | | | 4 | | 174.04 | 3.88 | | 0.02 | | -82.91 | | |
|  | | | | | PC2+PC2^2^+Year | | | 7 | | 174.11 | 3.96 | | 0.02 | | -79.73 | | |
|  | | | | | SMI+SMI^2^ | | | 4 | | 174.28 | 4.12 | | 0.02 | | -83.03 | | |
|  | | | | | Muscle+Year | | | 6 | | 175.19 | 5.04 | | 0.01 | | -81.36 | | |
|  | | | | | PC2+Year | | | 6 | | 175.20 | 5.04 | | 0.01 | | -81.36 | | |
|  | | | | | Muscle+Muscle^2^+Year | | | 7 | | 176.71 | 6.55 | | 0.01 | | -81.03 | | |
|  | | | | | Stage | | | 7 | | 177.66 | 7.51 | | 0.00 | | -81.51 | | |
|  | | | | | Year+Stage | | | 10 | | 180.43 | 10.27 | | 0.00 | | -79.57 | | |
|  | | | | | Fat+Sex+Stage+Year | | | 12 | | 184.56 | 14.41 | | 0.00 | | -79.36 | | |
|  | | | | | Fat+Fat^2^+Sex+Stage+Year | | | 13 | | 185.77 | 15.62 | | 0.00 | | -78.80 | | |
|  | | | | | PCV+Sex+Stage+(Sex × Stage) | | | 14 | | 187.25 | 17.09 | | 0.00 | | -78.37 | | |
|  | | | | | SMI+Sex+Stage+(Sex × Stage)+Time | | | 15 | | 189.25 | 19.10 | | 0.00 | | -78.18 | | |
|  | | | | | PCV+PCV^2^+Sex+Stage+(Sex × Stage) | | | 15 | | 189.61 | 19.46 | | 0.00 | | -78.36 | | |
|  | | | | | PC1+Sex+Stage+(Sex × Stage)+Year* | | | 17 | | 190.70 | 20.55 | | 0.00 | | -76.48 | | |
|  | | | | | Hb+Sex+Stage+(Sex × Stage)+Year | | | 17 | | 190.82 | 20.67 | | 0.00 | | -76.54 | | |
|  | | | | | SMI+SMI^2^+Sex+Stage+(Sex × Stage)+Time | | | 16 | | 191.62 | 21.47 | | 0.00 | | -78.16 | | |
|  | | | | | Hb+Hb^2^+Sex+Stage+(Sex × Stage)+Year | | | 18 | | 191.99 | 21.84 | | 0.00 | | -75.90 | | |
|  | | | | | PC1+PC1^2^+Sex+Stage+(Sex × Stage) | | | 18 | | 192.34 | 22.19 | | 0.00 | | -76.07 | | |
|  | | | | |  | | |  | |  |  | |  | |  | | |
| Number independent | | | | | Year+SMI+SMI^2^ | | | 7 | | 336.49 | 0.00 | | 0.28 | | -160.92 | | |
|  | | | | | Year+SMI | | | 6 | | 337.95 | 1.47 | | 0.13 | | -162.74 | | |
|  | | | | | Year+PC2+PC2^2^ | | | 7 | | 338.40 | 1.92 | | 0.11 | | -161.88 | | |
|  | | | | | Year+PC1 | | | 6 | | 339.15 | 2.66 | | 0.07 | | -163.33 | | |
|  | | | | | Year+PC1+PC1^2^ | | | 7 | | 339.82 | 3.33 | | 0.05 | | -162.59 | | |
|  | | | | | Year+Muscle | | | 6 | | 340.10 | 3.61 | | 0.05 | | -163.81 | | |
|  | | | | | Year+PC2 | | | 6 | | 340.12 | 3.63 | | 0.05 | | -163.82 | | |
|  | | | | | Year+Fat+Fat^2^ | | | 7 | | 340.28 | 3.80 | | 0.04 | | -162.82 | | |
|  | | | | | **Year** | | | 5 | | 340.47 | 3.99 | | 0.04 | | -165.07 | | |
|  | | | | | Year+Hb+Hb^2^ | | | 7 | | 340.64 | 4.16 | | 0.03 | | -163.00 | | |
|  | | | | | Year+Fat | | | 6 | | 340.93 | 4.44 | | 0.03 | | -164.22 | | |
|  | | | | | Year+Muscle+Muscle^2^ | | | 7 | | 341.05 | 4.57 | | 0.03 | | -163.21 | | |
|  | | | | | Year+SMI+SMI^2^+Sex+Stage+(Sex × Stage)+Time | | | 19 | | 341.17 | 4.68 | | 0.03 | | -149.24 | | |
|  | | | | | Year+PCV | | | 6 | | 341.30 | 4.81 | | 0.03 | | -164.41 | | |
|  | | | | | Year+Hb | | | 6 | | 342.39 | 5.90 | | 0.01 | | -164.95 | | |
|  | | | | | Year+PCV+PCV^2^ | | | 7 | | 342.60 | 6.12 | | 0.01 | | -163.98 | | |
|  | | | | | Year+SMI+Sex+Stage+(Sex × Stage)+Time | | | 18 | | 343.64 | 7.15 | | 0.01 | | -151.72 | | |
|  | | | | | Year+Stage | | | 10 | | 346.05 | 9.56 | | 0.00 | | -162.38 | | |
|  | | | | | Year+Fat+Sex+Stage | | | 12 | | 349.73 | 13.25 | | 0.00 | | -161.94 | | |
|  | | | | | Year+Fat+Fat^2^+Sex+Stage | | | 13 | | 350.35 | 13.87 | | 0.00 | | -161.09 | | |
|  | | | | | Year+PC1+Sex+Stage+(Sex × Stage) | | | 17 | | 351.88 | 15.39 | | 0.00 | | -157.07 | | |
|  | | | | | Year+PCV+Sex+Stage+(Sex × Stage) | | | 17 | | 353.48 | 16.99 | | 0.00 | | -157.87 | | |
|  | | | | | Year+PC1+PC1^2^+Sex+Stage+(Sex × Stage) | | | 18 | | 353.80 | 17.32 | | 0.00 | | -156.80 | | |
|  | | | | | Year+Hb+Sex+Stage+(Sex × Stage) | | | 17 | | 354.64 | 18.15 | | 0.00 | | -158.45 | | |
|  | | | | | Year+Hb+Hb^2^+Sex+Stage+(Sex × Stage) | | | 18 | | 355.12 | 18.63 | | 0.00 | | -157.46 | | |
|  | | | | | Year+PCV+PCV^2^+Sex+Stage+(Sex × Stage) | | | 18 | | 355.58 | 19.10 | | 0.00 | | -157.69 | | |
|  | | | | | Null | | | 2 | | 361.79 | 25.30 | | 0.00 | | -178.86 | | |
|  | | | | | Stage | | | 7 | | 363.04 | 26.55 | | 0.00 | | -174.20 | | |
|  |  |  | |  | |  |  |  |  |  |  |  |  |  |  |  |  |
